# Sleep Architecture Alterations Following High‐Dose Steroid Pulse Therapy: A Pilot Study Using a Portable Electroencephalogram‐Based Device

**DOI:** 10.1002/npr2.70071

**Published:** 2025-11-09

**Authors:** Hiroki Endo, Yuki Shigetsura, Misaki Chahara, Keisuke Kido, Etsuro Nakanishi, Sakiho Ueda, Kimitoshi Kimura, Akira Kuzuya, Hirotsugu Kawashima, Riki Matsumoto, Masahiro Tsuda, Shunsaku Nakagawa, Tomohiro Terada

**Affiliations:** ^1^ Department of Clinical Pharmacology and Therapeutics Kyoto University Hospital Kyoto Japan; ^2^ Department of Neurology, Graduate School of Medicine Kyoto University Kyoto Japan; ^3^ Department of Psychiatry, Graduate School of Medicine, Kyoto University Kyoto Japan

**Keywords:** high‐dose steroid pulse therapy, portable EEG‐based device, sleep architecture

## Abstract

**Background:**

Sleep disturbance is a common side effect of high‐dose steroid pulse therapy (SPT). However, in clinical settings, the impact of this therapy on sleep architecture has not been objectively studied.

**Objective:**

This study aimed to investigate changes in sleep architecture associated with high‐dose SPT administration in patients with neuroimmunological disorders.

**Methods:**

A prospective cohort study was conducted involving six hospitalized patients with neuroimmunological disorders who were administered intravenous methylprednisolone (1000 mg/day for 3 days). Objective sleep parameters were assessed on days 0, 1, and 3 using a SleepGraph, a validated portable electroencephalography (EEG) device. Subjective sleep was evaluated using the Insomnia Severity Index (ISI), Pittsburgh Sleep Quality Index (PSQI), sleep diary, and Likert scale.

**Results:**

Rapid eye movement (REM) sleep significantly decreased from 71.2 ± 36.6 min on day 0 to 9.4 ± 8.3 min on day 1 and remained low on day 3 (16.5 ± 16.0 min). Total sleep time was also reduced, while non‐REM (NREM) sleep remained relatively stable. Two patients who received lemborexant showed partial improvement in their sleep parameters. ISI and PSQI scores did not change significantly, possibly owing to the short observation period.

**Conclusion:**

These findings suggest that high‐dose SPT can markedly reduce REM sleep. Moreover, portable EEG devices could be a practical approach for monitoring steroid‐induced sleep alterations. Our findings provide clinical evidence that may help to deepen the objective understanding of sleep during SPT.

## Abstract 2

High‐dose steroid pulse therapy markedly reduces total sleep time, REM, and NREM sleep, as objectively demonstrated for the first time using a portable EEG‐based device.

## Graphical abstract

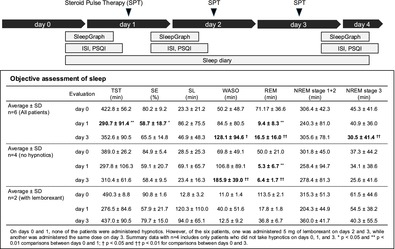

## 1 Introduction

High‐dose steroid pulse therapy (SPT) is a standard treatment for neuroimmunological disorders [1, 2], with 1000 mg/day of methylprednisolone for 3 days commonly used globally [3, 4, 5]. Sleep disturbance is a frequent adverse effect of this treatment, occurring in ~70% of patients [6, 7]. However, in clinical practice, no standard treatment has been established for steroid‐induced sleep disturbance. Hypnotics are often administered empirically, but the most effective agent remains unclear. Furthermore, while the effects of corticosteroids on sleep architecture have been demonstrated in rodent models [8], their impact on human sleep architecture in real‐world clinical settings using electroencephalography (EEG)‐based devices has yet to be elucidated. A better understanding of sleep architecture may offer clinically relevant insights into symptoms, comorbidities, and appropriate hypnotics.

Recently, some portable EEG‐based devices have offered a low‐burden alternative with high accuracy and reliability [9, 10, 11, 12, 13]. The present study aimed to investigate changes in sleep architecture following SPT in patients with neuroimmunological disorders using a portable EEG‐based device. Clarifying the alterations in sleep parameters during SPT may help guide the appropriate use of hypnotics to improve patients' quality of life, while also providing insights into the neuropsychopharmacological mechanisms of steroid‐induced sleep disturbance.

## 2 Methods

### 2.1 Study Participants and Outcome Measurements

Six hospitalized patients (≥ 18 years) receiving high‐dose SPT (methylprednisolone 1000 mg/body/day for 3 days) for neuroimmunological disorders at Kyoto University Hospital were enrolled between June 2024 and May 2025. Written informed consent was obtained from all participants by the attending physicians or pharmacists. Exclusion criteria included contraindications to portable EEG (e.g., pacemaker use), inability to perform pretreatment evaluation or complete self‐assessments, history of epilepsy, pregnancy or lactation, history of severe drug allergies, high risk of suicide, and regular hypnotic use (unless started after therapy). High‐dose SPT was administered in the morning at a fixed dose of 1000 mg/day, which corresponded to 10–21 mg/kg/day when adjusted for the patients' body weights.

Demographic, clinical, and sleep‐related data were collected, along with residual serum samples during the course of SPT, specifically on days 0, 1, and 3. Serum allopregnanolone, a neurosteroid implicated in steroid‐induced sleep disturbance [14, 15], was measured using a commercial enzyme‐linked immunosorbent assay (ELISA) kit (Abcam, Cambridge, UK). Cortisol and adrenocorticotropic hormone (ACTH) levels were also measured. Serum cortisol was quantified using the AIA‐Pack CL Cortisol assay on the AIA‐CL2400 analyzer (Tosoh Bioscience, Tokyo, Japan) with the chemiluminescent enzyme immunoassay (CLEIA) method. ACTH was measured using the Elecsys ACTH assay (Roche Diagnostics K.K., Tokyo, Japan) on the cobas 8000 e801 analyzer (Roche Diagnostics K.K., Tokyo, Japan) based on the electrochemiluminescence immunoassay (ECLIA) method.

### 2.2 Objective or Subjective Assessment of Sleep

Objective sleep was assessed using the SleepGraph (Proassist, Osaka, Japan) before (day 0) and on days 1 and 3 of SPT. The SleepGraph is a portable, EEG‐based device that employs four surface electrodes placed at the following sites: the right frontal scalp (Fp2), the infra‐orbital area beneath the left eye, the right mandibular angle, and the left mastoid (M1). The Fp2‐to‐M1 derivation simultaneously captures EEG activity and the right electro‐oculogram (EOG), whereas the infra‐orbital‐to‐M1 derivation records the left EOG. Chin muscle tone is measured via an electromyogram channel obtained between the right mandibular electrode and M1.

To minimize patient burden and avoid disturbing natural sleep, we employed the telemetry EEG‐based device SleepGraph. Furthermore, SleepGraph provides a sufficient number of channels to enable manual scoring. The SleepGraph is an approved medical device in Japan (approval number: 231AHBZX00001000). Sleep data were scored manually according to the standard criteria in 30‐s epochs as Stage W, Stage N1 + N2 (NREM N1–2), Stage N3 (NREM N3), or Stage R (REM). Subjective sleep was evaluated using a sleep diary [16], 10‐point Likert scales for sleep quality and daytime impairment, and validated Japanese versions of the Insomnia Severity Index (ISI‐J) [17] and Pittsburgh Sleep Quality Index (PSQI‐J) [18]. The sleep diary tracked bedtimes, sleep latency (SL), number and duration of awakenings, and final wake‐up times throughout the study.

### 2.3 Statistical Analysis

Statistical analyses were conducted using R software (v4.2.1; R Foundation for Statistical Computing, Vienna, Austria) with the *nlme* and *emmeans* packages. A linear mixed‐effects model, specifying day as a fixed effect and subject as a random effect, was used to compare days 0, 1, and 3 using Tukey's test. Significance was set at 5%. A post hoc power analysis using the *pwr* package was performed based on a two‐sided paired t‐test (α = 0.05), estimating power from the observed effect size (Cohen's *d*) and sample size (*n* = 6).

## 3 Results

### 3.1 Patient Characteristics

The characteristics of the six patients included in this study are summarized in Table 1. The mean age of the six patients was 46 years, with an equal distribution of males and females. No hypnotics were used on days 0 and 1; however, 5 mg of lemborexant was administered to Patient 4 on days 2 and 3, and to Patient 5 on day 3. One patient exhibited a 3% oxygen desaturation index (ODI‐3) > 15. No psychiatric symptoms such as steroid‐induced depression were clinically observed during steroid treatment, although standardized assessment tools were not applied.

**TABLE 1:** Patient characteristics.

Patient	Sex	Age	BMI (kg/m^2^)	Underlying disease	Pre‐pulse hospitalization period (days)	ALT (IU/L)	Serum creatinine (mg/dL)	CRP (mg/dL)	ODI‐3	Regular medications at admission (per day)
No. 1	Male	50s	29.12	Suspected autoimmune disorder with anti‐NAE antibody positivity	5	25	1.11	< 0.10	19.53	Amezinium 10 mg, Droxidopa 400 mg, Fludrocortisone 0.05 mg, Goreisan 7.5 g, Levothyroxine 75 μg, Magnesium oxide 990 mg, Midodrine 4 mg, Vibegron 50 mg
No. 2	Female	50s	25.85	Suspected autoimmune myelitis	3	24	0.59	0.14	8.14	None
No. 3	Male	50s	24.82	Suspected post‐COVID‐19 myelitis	10	41	0.72	0.13	14.41	Amlodipine 5 mg, Febuxostat 20 mg, Mecobalamin 1500 μg, Limaprost alfadex 15 μg
No. 4	Male	20s	18.60	Guillain–Barré syndrome	57	22	0.65	< 0.10	2.56	Bilastine 20 mg
No. 5	Female	30s	21.64	Anti‐SRP antibody‐positive myopathy	4	25	0.63	< 0.10	6.59	None
No. 6	Female	50s	19.54	Atypical multiple sclerosis	3	8	0.48	< 0.10	1.70	None

*Note:* ODI‐3 defined as the 3% oxygen desaturation index per hour measured by pulse oximeter (PULSOX‐500i, Konica Minolta Sensing, Osaka, Japan). Biochemical parameters were measured on day 0.

Abbreviations: ALT, alanine aminotransferase; BMI, body mass index; CRP, C‐reactive protein; Goreisan, a herbal medicine commonly used to manage fluid imbalance and edema; N/A, not available (some values were missing for certain patients); NAE, NH₂‐terminal of α‐enolase; ODI‐3, 3% oxygen desaturation index; SRP, signal recognition particle.

### 3.2 Alterations in Sleep Architecture Assessed Using a Portable EEG‐Based Device

The data measurement status was stable over each recording night, with only minimal artifacts. The objective sleep parameters are summarized in Table 2. The individual patient‐level objective sleep parameters are provided in Table S1, and a representative recording is shown in Figure S1. In all patients (*n* = 6), total sleep time (TST) significantly decreased from day 0 (422.8 ± 56.2 min) to day 1 (290.7 ± 91.4 min; *p* < 0.01) and remained reduced on day 3 (352.6 ± 90.5 min). REM sleep also decreased significantly on day 1 (9.4 ± 8.3 min; *p* < 0.01) and remained lower on day 3 (16.5 ± 16.0 min) compared with day 0 (71.2 ± 36.6 min). SL and wake after sleep onset (WASO) showed increasing trends after day 0, although the differences were not statistically significant. Stage N3 duration was significantly reduced on day 3 compared to that on day 0 (*p* < 0.01). In patients who did not receive hypnotics (*n* = 4), REM sleep was significantly reduced on both day 1 (5.3 ± 6.7 min; *p* < 0.01) and day 3 (6.4 ± 1.7 min; *p* < 0.01) compared to day 0 (50.0 ± 21.0 min). WASO was also significantly prolonged on day 3 (185.9 ± 39.0 min) versus day 0 (69.8 ± 49.1 min; *p* < 0.01). Two patients who received lemborexant (5 mg) on day 3 showed improved TST (276.5 ± 84.6 min on day 1 to 437.0 ± 90.5 min on day 3) and REM sleep (17.8 ± 1.8 min to 36.8 ± 6.7 min), though these did not return to baseline levels.

**TABLE 2:** Objective assessment of sleep using portable electroencephalography.

	Evaluation	TST (min)	SE (%)	SL (min)	WASO (min)	REM (min)	NREM stage 1 + 2 (min)	NREM stage 3 (min)	SO	Final awakening
Average ± SD *n* = 6 (all patients)	Day 0	422.8 ± 56.2	80.2 ± 9.2	23.3 ± 21.2	50.2 ± 48.7	71.17 ± 36.6	306.4 ± 42.3	45.3 ± 41.6	21:46	5:41
	Day 1	290.7 ± 91.4**	58.7 ± 18.7*	86.2 ± 75.5	84.5 ± 80.5	9.4 ± 8.3**	240.3 ± 81.0	40.9 ± 36.0	23:08	5:23
	Day 3	352.6 ± 90.5	65.5 ± 14.8	46.9 ± 48.3	128.1 ± 94.6^†^	16.5 ± 16.0^††^	305.6 ± 78.1	30.5 ± 41.4^††^	22:19	6:20
Average ± SD *n* = 4 (no hypnotics)	Day 0	389.0 ± 26.2	84.9 ± 5.4	28.5 ± 25.3	69.8 ± 49.1	50.0 ± 21.0	301.8 ± 45.0	37.3 ± 44.2	21:26	5:06
	Day 1	297.8 ± 106.3	59.1 ± 20.7	69.1 ± 65.7	106.8 ± 89.1	5.3 ± 6.7**	258.4 ± 94.7	34.1 ± 38.6	22:13	4:58
	Day 3	310.4 ± 61.6	58.4 ± 9.5	23.4 ± 16.3	185.9 ± 39.0^††^	6.4 ± 1.7^††^	278.4 ± 81.3	25.6 ± 41.6	21:31	5:48
Average ± SD *n* = 2 (with lemborexant)	Day 0	490.3 ± 8.8	90.8 ± 1.6	12.8 ± 3.2	11.0 ± 1.4	113.5 ± 2.1	315.3 ± 51.3	61.5 ± 44.6	22:28	6:49
	Day 1	276.5 ± 84.6	57.9 ± 21.7	120.3 ± 110.0	40.0 ± 51.6	17.8 ± 1.8	204.3 ± 44.9	54.5 ± 38.2	1:00	6:17
	Day 3	437.0 ± 90.5	79.7 ± 15.0	94.0 ± 65.1	12.5 ± 9.2	36.8 ± 6.7	360.0 ± 41.7	40.3 ± 55.5	23:56	7:26

*Note:* Summary statistics related to time were converted into circular data for analysis. Subsequently, to facilitate interpretation, the times were back transformed into standard clock times. On days 0 and 1, none of the patients were administered hypnotics. However, of the six patients, one was administered 5 mg of lemborexant on days 2 and 3, while another was administered the same dose on day 3. Summary data with *n* = 4 includes only patients who did not take hypnotics on days 0, 1, and 3. *p < 0.05 and **p < 0.01 comparisons between days 0 and 1; ^†^ *p* < 0.05 and ^††^ *p* < 0.01 for comparisons between days 0 and 3.

Abbreviations: NREM, non‐rapid eye movement; REM, rapid eye movement sleep; SD, standard deviation; SE, sleep efficiency; SL, sleep latency; SO, sleep onset; TST, total sleep time; WASO, wake after sleep onset.

### 3.3 Changes in Subjective Sleep Measures

Table 3 shows changes in subjective sleep parameters across days 0, 1, and 3. The individual patient‐level subjective sleep parameters are provided in Table S2. TST significantly decreased from day 0 (448.3 ± 30.8 min) to day 1 (276.2 ± 114.7 min; *p* < 0.01). ISI and PSQI scores showed no significant changes over time. SL and WASO tended to increase on day 1, but without statistical significance. Likert‐scale ratings for sleep quality and daytime impairment also remained stable, though a slight increase in impairment was reported on day 3.

**TABLE 3:** Subjective assessment of sleep.

	Evaluation	ISI	PSQI	Sleep diary TST (min)	Sleep diary			Sleep quality Likert scale	Impairment Likert scale
					SL (min)	WASO (min)	WASO (times)		
Average ± SD *n* = 6 (all patients)	Day 0	8.0 ± 4.9	5.3 ± 3.4	448.3 ± 30.8	33.3 ± 28.6	40.8 ± 30.7	1.8 ± 1.2	6.0 ± 2.3	1.7 ± 0.8
	Day 1	11.2 ± 5.3	5.8 ± 2.6	276.2 ± 114.7**	95.0 ± 95.2	71.3 ± 104.5	1.5 ± 1.4	6.0 ± 3.1	1.8 ± 1.2
	Day 3	11.3 ± 7.2	6.5 ± 3.7	332.5 ± 125.6	54.2 ± 36.1	91.7 ± 114.4	1.0 ± 0.6	5.5 ± 2.7	2.3 ± 1.5
Average ± SD *n* = 4 (no hypnotics)	Day 0	9.3 ± 5.1	4.8 ± 3.2	441.3 ± 29.6	41.3 ± 33.3	53.8 ± 27.5	2.3 ± 1.0	5.5 ± 2.6	2.0 ± 0.8
	Day 1	10.3 ± 5.7	5.5 ± 2.5	273.0 ± 142.8	93.8 ± 102.7	80.8 ± 126.5	1.8 ± 1.5	5.0 ± 3.5	2.3 ± 1.3
	Day 3	12.0 ± 6.5	5.5 ± 2.4	292.5 ± 129.9	47.5 ± 12.6	118.8 ± 136.2	1.0 ± 0.8	5.3 ± 3.1	3.0 ± 1.4
Average ± SD *n* = 2 (with lemborexant)	Day 0	5.5 ± 5.0	6.5 ± 5.0	462.5 ± 38.9	17.5 ± 3.5	15.0 ± 21.2	1.0 ± 1.4	7.0 ± 1.4	1.0 ± 0.0
	Day 1	13.0 ± 5.7	6.5 ± 3.5	282.5 ± 67.2	97.5 ± 116.7	52.5 ± 74.3	1.0 ± 1.4	8.0 ± 0.0	1.0 ± 0.0
	Day 3	10.0 ± 11.3	8.5 ± 6.4	412.5 ± 95.5	67.5 ± 74.3	37.5 ± 31.8	1.0 ± 0.0	6.0 ± 2.8	1.0 ± 0.0

*Note:* The participants rated their perceived sleep quality and daytime impairment on a 10‐point Likert scale (1 = best, 10 = worst). On days 0 and 1, none of the patients was administered hypnotics. However, of the six patients, one was administered 5 mg of lemborexant on days 2 and 3, while another was administered the same dose on day 3. Summary data with *n* = 4 includes only patients who did not take any hypnotics on days 0, 1, and 3. Statistical analysis was performed using a linear mixed‐effects model with “day” as a fixed effect and “subject” as a random effect. To evaluate the differences in measurements across days while accounting for individual variability, multiple comparisons were conducted using Tukey's method.

Abbreviations: ISI, Insomnia Severity Index; PSQI, Pittsburgh Sleep Quality Index; SD, standard deviation; SL, sleep latency; TST, total sleep time; WASO, wake after sleep onset.

** *p* < 0.01 between days 0 and 1.

In patients not receiving hypnotics (*n* = 4), TST decreased similarly on day 1 (273.0 ± 142.8 min) compared with day 0 (441.3 ± 29.6 min), while other parameters remained stable. The two patients who received lemborexant showed increased TST on day 3 (412.5 ± 95.5 min vs. 282.5 ± 67.2 min on day 1). Apart from the administration of lemborexant, no changes in concomitant medications, physical conditions, or sleep environments that could have affected sleep were observed in these patients.

### 3.4 Serum Hormone and Neurosteroid Levels

Serum hormone and neurosteroid levels assessed in five patients with baseline data showed serum cortisol levels of 7.3–13.4 μg/dL, ACTH of 9.0–74.0 pg/mL, and allopregnanolone of 55.2–118.5 pg/mL. Post‐treatment samples (*n* = 4) showed marked suppression of cortisol (0.2–3.7 μg/dL) and ACTH (< 1.5–43.6 pg/mL), while allopregnanolone levels varied (58.6–165.0 pg/mL) without a consistent pattern. Individual data are presented in Table 4.

**TABLE 4:** Hormones related to the hypothalamic–pituitary–adrenal (HPA) axis and allopregnanolone.

Patient	Pre‐steroid pulse blood sampling day	Serum cortisol (μg/dL)	Serum ACTH (pg/mL)	Serum allopregnanolone (pg/mL)	Post‐steroid pulse blood sampling day	Serum cortisol (μg/dL)	Serum ACTH (pg/mL)	Serum allopregnanolone (pg/mL)
No. 1	Day −2	9.3	64.8	55.2	Day 12	0.5	< 1.5	58.6
No. 2	Day 0	7.3	9.0	75.5	N/A	N/A	N/A	N/A
No. 3	Day 0	13.4	74.0	118.5	Day 5	2.6	26.0	60.7
No. 4	Day 0	10.4	38.2	109.1	Day 6	3.7	43.6	132.3
No. 5	Day 0	11.4	31.2	115.2	Day 12	0.2	1.6	165.0
No. 6	N/A	N/A	N/A	N/A	N/A	N/A	N/A	N/A

*Note:* Day 1 was defined as the first day of steroid pulse therapy, with the preceding day designated as Day 0.

Abbreviations: ACTH, adrenocorticotropic hormone; N/A, blood sampling was not performed.

## 4 Discussion

To our knowledge, this study is the first clinical report to evaluate steroid‐induced sleep disturbance using a portable EEG‐based device. As expected, reduced TST was objectively confirmed by the sleep study. High‐dose SPT markedly reduced REM sleep in this study. Short‐term REM reduction is linked to negative mood states, amygdala hyperactivity, impaired anterior cingulate connectivity, memory deficits, anxiety, irritability, impaired concentration, and stress‐related prefrontal dysfunction [19, 20, 21, 22]. Furthermore, long‐term REM loss is associated with increased mortality and dementia risk [23, 24], highlighting the importance of REM preservation during treatment. As SPT is often followed by longer oral regimens, strategies to preserve REM sleep may be clinically important. Notably, in two patients, lemborexant improved almost all sleep parameters. This observation is consistent with prior evidence from rodent models and clinical trials in humans demonstrating that lemborexant increases both REM and NREM sleep. These findings suggest favorable effects of lemborexant on sleep parameters [25, 26]. Apart from the administration of lemborexant, no changes in concomitant medications, physical conditions, or sleep environments that could have affected sleep were observed, suggesting that the improvements in sleep parameters were attributable to lemborexant. However, the varying effects of hypnotics underscore the need for further research on optimal agents [27, 28]. These findings provide important insights toward elucidating the clinical picture of SPT‐induced sleep disturbance, and understanding changes in sleep parameters alongside patients' clinical symptoms. As such knowledge may facilitate evidence‐based strategies, such as the early detection of REM sleep, NREM sleep, and TST reduction during SPT and the rational selection of hypnotics like orexin receptor antagonists, to mitigate sleep disruption and improve patient outcomes. Furthermore, they contribute to the neuropsychopharmacological field by demonstrating a clear association between corticosteroid exposure and objectively measured changes in sleep parameters.

In this study, some patients exhibited near‐complete loss of REM sleep. Given the high inter‐scorer reliability of SleepGraph, these changes likely reflect true alterations rather than scoring artifacts [29]. Obstructive sleep apnea syndrome (OSAS) may cause sleep fragmentation and reduced REM sleep [30]. One patient had an ODI‐3 ≥ 15, indicating possible OSAS influence, while others did not, suggesting limited impact.

The mechanism underlying the reduction of REM sleep associated with steroid‐induced insomnia remains unclear; however, several hypotheses can be considered. The occurrence of REM sleep is regulated by a flip‐flop switch between the ventrolateral preoptic nucleus and brainstem arousal centers that include orexin‐producing neurons. Orexin neurons have been reported to express glucocorticoid receptors [31], suggesting that steroids may influence orexin activity. Furthermore, chemogenetic activation of orexin neurons has been shown to shorten total REM sleep time [32], raising the possibility that steroid exposure may have contributed to the reduction of REM sleep observed in the present study. In fact, the finding that an orexin receptor antagonist improved the REM sleep shortening induced by SPT provides additional support for this hypothesis.

Moreover, steroids are known to suppress nocturnal melatonin synthesis [33], and insufficient nocturnal melatonin secretion has been reported to shorten REM sleep [34]. Thus, it is also conceivable that steroids reduce REM sleep via suppression of melatonin secretion. In any case, these remain hypotheses, and further studies are warranted to elucidate the underlying mechanisms.

Subjective tools such as the ISI and PSQI exhibited limited sensitivity to short‐term changes, which may explain the discrepancy with sleep studies. However, as these tools were originally designed to assess sleep for 2 weeks or more [17, 18], simpler tools such as portable EEG‐based devices or sleep diaries may be more suitable for short‐term clinical monitoring.

Although the mechanisms of steroid‐induced sleep disturbance remain unclear, they may involve hypothalamic–pituitary–adrenal axis hyperactivity or reduced levels of neurosteroids, such as allopregnanolone [6]. The lack of a consistent trend in allopregnanolone levels suggests that peripheral measures may not fully capture central neurosteroid dynamics, or there may be limitations in sample timing and consistency. Further mechanistic studies are warranted.

The first limitation of this report includes the small sample size, single‐center design, background heterogeneity, and the absence of a non‐steroid control group, as this was a single‐arm study. Consequently, the disease‐related alternation in sleep could not be specifically evaluated. For example, Guillain‐Barré syndrome has been reported to be associated with sleep disturbances, particularly due to sleep‐disordered breathing and fragmented sleep parameters [35].

However, a post hoc power analysis confirmed the large effects observed (e.g., REM sleep; Cohen's *d* = 1.90, power = 0.96). The second limitation is the use of a portable EEG‐based device instead of standard polysomnography. However, the SleepGraph system has demonstrated high consistency with polysomnography [36, 37] and high inter‐scorer reliability in diverse sleep research [29], supporting its validity. Further studies should investigate underlying mechanisms and evaluate long‐term interventions to mitigate steroid‐induced sleep disturbance.

## 5 Conclusion

Our findings provide novel clinical evidence that high‐dose SPT objectively and markedly reduces TST, REM sleep, and NREM sleep. These results highlight a previously underrecognized impact of corticosteroid therapy on patient sleep. The portable EEG‐based device offers a feasible approach for detecting steroid‐induced sleep alterations. Further studies are required to clarify the underlying mechanisms and to identify effective interventions to preserve sleep architecture during corticosteroid therapy.

## Author Contributions

Study design and conceptualization were performed by H.E., Y.S., and K. Kido. Data were acquired by H.E., Y.S., and M.C. Sleep data were scored, and representative data were selected by K. Kido. Data analysis and interpretation were performed by H.E., Y.S., and K. Kido. H.E. and Y.S. drafted the manuscript. Critical revision of the manuscript for important intellectual content was performed by H.E., Y.S., M.C., K. Kido, H.K., E.N., S.U., K. Kimura, A.K., R.M., M.T., S.N., and T.T. All authors reviewed and approved the final manuscript and accept full responsibility for its content, including the investigation and resolution of any issues concerning its accuracy and integrity.

## Ethics Statement

This study was conducted in accordance with the principles of the Declaration of Helsinki and was approved by the Ethics Committee of Kyoto University Graduate School of Medicine, School of Medicine, and Faculty of Medicine Hospital (Approval Number, R4438).

## Consent

Written informed consent was obtained from all participants before their study inclusion.

## Conflicts of Interest

The authors declare no conflicts of interest.

## Supporting information


**Figure S1:** npr270071‐sup‐0001‐Figure S1.docx.

**Table S1:** npr270071‐sup‐0002‐TableS1.xlsx.

**Table S2:** npr270071‐sup‐0003‐TableS2.docx.

## Data Availability

All de‐identified raw data are provided in Tables S1 and S2. Regarding the electroencephalography, electro‐oculography, and electromyography files in EDF format, although the risk of immediate personal identification is low, complete anonymization cannot be guaranteed. Therefore, only aggregated data are publicly available as Supporting Information, whereas the raw EDF files are available from the corresponding author upon reasonable request.

## References

[1] D. B. Sanders , G. I. Wolfe , M. Benatar , et al., “International Consensus Guidance for Management of Myasthenia Gravis: Executive Summary,” Neurology 87, no. 4 (2016): 419–425. 27358333 10.1212/WNL.0000000000002790 PMC4977114

[2] S. E. Leonhard , M. R. Mandarakas , F. A. A. Gondim , et al., “Diagnosis and Management of Guillain‐Barré Syndrome in Ten Steps,” Nature Reviews Neurology 15, no. 11 (2019): 671–683. 31541214 10.1038/s41582-019-0250-9 PMC6821638

[3] T. Kümpfel , K. Giglhuber , O. Aktas , et al., “Update on the Diagnosis and Treatment of Neuromyelitis Optica Spectrum Disorders (NMOSD)—Revised Recommendations of the Neuromyelitis Optica Study Group (NEMOS). Part II: Attack Therapy and Long‐Term Management,” Journal of Neurology 271, no. 1 (2024): 141–176. 37676297 10.1007/s00415-023-11910-z PMC10770020

[4] B. Yamout , M. Al‐Jumah , M. A. Sahraian , et al., “Consensus Recommendations for Diagnosis and Treatment of Multiple Sclerosis: 2023 Revision of the MENACTRIMS Guidelines,” Multiple Sclerosis and Related Disorders 83 (2024): 105435. 38245998 10.1016/j.msard.2024.105435

[5] H. Murai , “The Japanese Clinical Guidelines 2022 for Myasthenia Gravis and Lambert‐Eaton Myasthenic Syndrome: An Overview,” Brain and Nerve 76, no. 1 (2024): 7–12. 38191133 10.11477/mf.1416202551

[6] J. L. Cole , “Steroid‐Induced Sleep Disturbance and Delirium: A Focused Review for Critically Ill Patients,” Federal Practitioner 37, no. 6 (2020): 260–267. 32669778 PMC7357890

[7] T. Fujita , Y. Fukuyama , N. Misumi , and T. Nishino , “Investigation on the Onset of Insomnia Resulting From the Introduction of Steroids in Collagenosis Internal Medicine,” Japanese Journal of Hospital Pharmacy 53, no. 10 (2017): 1257–1260.

[8] T. Kato , G. Okawa , K. F. Tanaka , and Y. Mitsukura , “Dexamethasone Induces Sleep Disturbance in a Dose‐Dependent Manner in Mice,” PLoS One 18, no. 12 (2023): e0296028. 38117835 10.1371/journal.pone.0296028 PMC10732373

[9] S. Nakajima , Y. Kaneko , N. Fujii , et al., “Transdiagnostic Association Between Subjective Insomnia and Depressive Symptoms in Major Psychiatric Disorders,” Frontiers in Psychiatry 14 (2023): 1114945. 37168089 10.3389/fpsyt.2023.1114945 PMC10165079

[10] M. R. Pretel , V. Vidal , D. Kienigiel , C. Forcato , and R. Ramele , “A Low‐Cost and Open‐Hardware Portable 3‐Electrode Sleep Monitoring Device,” HardwareX 19 (2024): e00553. 39099722 10.1016/j.ohx.2024.e00553 PMC11295469

[11] A. Kawamura , H. Kadotani , M. Suzuki , M. Uchiyama , N. Yamada , and K. Kuriyama , “Objective Evaluation of Major Depressive Disorder Using Sleep Electroencephalography Measured by an In‐Home Portable One‐Channel Device: A Preliminary Study,” Sleep & Breathing 29, no. 2 (2025): 165. 40259132 10.1007/s11325-025-03329-9 PMC12011895

[12] C. Han , Z. Zhang , Y. Lin , et al., “Monitoring Sleep Quality Through Low α‐Band Activity in the Prefrontal Cortex Using a Portable Electroencephalogram Device: Longitudinal Study,” Journal of Medical Internet Research 27 (2025): e67188. 40063935 10.2196/67188 PMC11933759

[13] K. Kawai , K. Iwamoto , S. Miyata , et al., “Comparison of Polysomnography, Single‐Channel Electroencephalogram, Fitbit, and Sleep Logs in Patients With Psychiatric Disorders: Cross‐Sectional Study,” Journal of Medical Internet Research 25 (2023): e51336. 38090797 10.2196/51336 PMC10753421

[14] A. R. Genazzani , F. Petraglia , F. Bernardi , et al., “Circulating Levels of Allopregnanolone in Humans: Gender, Age, and Endocrine Influences,” Journal of Clinical Endocrinology and Metabolism 83, no. 6 (1998): 2099–2103. 9626145 10.1210/jcem.83.6.4905

[15] H. J. Stürenburg , U. Fries , and K. Kunze , “Glucocorticoids and Anabolic/Androgenic Steroids Inhibit the Synthesis of GABAergic Steroids in Rat Cortex,” Neuropsychobiology 35, no. 3 (1997): 143–146. 9170119 10.1159/000119335

[16] C. E. Carney , D. J. Buysse , S. Ancoli‐Israel , et al., “The Consensus Sleep Diary: Standardizing Prospective Sleep Self‐Monitoring,” Sleep 35, no. 2 (2012): 287–302. 22294820 10.5665/sleep.1642 PMC3250369

[17] C. H. Bastien , A. Vallières , and C. M. Morin , “Validation of the Insomnia Severity Index as an Outcome Measure for Insomnia Research,” Sleep Medicine 2, no. 4 (2001): 297–307. 11438246 10.1016/s1389-9457(00)00065-4

[18] Y. Doi , M. Minowa , M. Uchiyama , et al., “Psychometric Assessment of Subjective Sleep Quality Using the Japanese Version of the Pittsburgh Sleep Quality Index (PSQI‐J) in Psychiatric Disordered and Control Subjects,” Psychiatry Research 97, no. 2–3 (2000): 165–172. 11166088 10.1016/s0165-1781(00)00232-8

[19] R. W. Glosemeyer , S. Diekelmann , W. Cassel , et al., “Selective Suppression of Rapid Eye Movement Sleep Increases Next‐Day Negative Affect and Amygdala Responses to Social Exclusion,” Scientific Reports 10, no. 1 (2020): 17325. 33057210 10.1038/s41598-020-74169-8 PMC7557922

[20] S. J. Casey , L. C. Solomons , J. Steier , et al., “Slow Wave and REM Sleep Deprivation Effects on Explicit and Implicit Memory During Sleep,” Neuropsychology 30, no. 8 (2016): 931–945. 27797541 10.1037/neu0000314

[21] J. Cedernaes , F. H. Rångtell , E. K. Axelsson , et al., “Short Sleep Makes Declarative Memories Vulnerable to Stress in Humans,” Sleep 38, no. 12 (2015): 1861–1868. 26158890 10.5665/sleep.5228 PMC4667381

[22] W. Dement , “The Effect of Dream Deprivation,” Science 131, no. 3415 (1960): 1705–1707. 13815805 10.1126/science.131.3415.1705

[23] E. B. Leary , K. T. Watson , S. Ancoli‐Israel , et al., “Association of Rapid Eye Movement Sleep With Mortality in Middle‐Aged and Older Adults,” JAMA Neurology 77, no. 10 (2020): 1241–1251. 32628261 10.1001/jamaneurol.2020.2108 PMC7550971

[24] M. P. Pase , J. J. Himali , N. A. Grima , et al., “Sleep Architecture and the Risk of Incident Dementia in the Community,” Neurology 89, no. 12 (2017): 1244–1250. 28835407 10.1212/WNL.0000000000004373 PMC5606917

[25] C. T. Beuckmann , T. Ueno , M. Nakagawa , M. Suzuki , and S. Akasofu , “Preclinical In Vivo Characterization of Lemborexant (E2006), a Novel Dual Orexin Receptor Antagonist for Sleep/Wake Regulation,” Sleep 42, no. 6 (2019): zsz076. 30923834 10.1093/sleep/zsz076 PMC6559177

[26] M. Moline , G. Zammit , J. Y. Cheng , C. Perdomo , D. Kumar , and D. Mayleben , “Comparison of the Effect of Lemborexant With Placebo and Zolpidem Tartrate Extended Release on Sleep Architecture in Older Adults With Insomnia Disorder,” Journal of Clinical Sleep Medicine 17, no. 6 (2021): 1167–1174. 33590823 10.5664/jcsm.9150 PMC8314653

[27] F. M. R. de Mendonça , G. P. R. R. de Mendonça , L. C. Souza , et al., “Benzodiazepines and Sleep Architecture: A Systematic Review,” CNS & Neurological Disorders Drug Targets 22, no. 2 (2023): 172–179. 34145997 10.2174/1871527320666210618103344

[28] S. Nakamura , H. Yamadera , H. Suzuki , and S. Endo , “Chronobiological Research on Antidepressant Drugs: The Effect of the Antidepressant Drugs, Trazodone and Imipramine on the Circadian Rhythm Using Electroencephalography in Healthy Volunteers,” Journal of Nippon Medical School 69, no. 3 (2002): 262–267. 12068317 10.1272/jnms.69.262

[29] S. Nonoue , M. Mashita , S. Haraki , et al., “Inter‐Scorer Reliability of Sleep Assessment Using EEG and EOG Recording System in Comparison to Polysomnography,” Sleep and Biological Rhythms 15 (2017): 39–48.

[30] M. R. Bonsignore , E. Mazzuca , P. Baiamonte , B. Bouckaert , W. Verbeke , and D. A. Pevernagie , “REM Sleep Obstructive Sleep Apnoea,” European Respiratory Review 33, no. 171 (2024): 230166. 38355150 10.1183/16000617.0166-2023 PMC10865098

[31] L. A. Grafe , A. Cornfeld , S. Luz , R. Valentino , and S. Bhatnagar , “Orexins Mediate Sex Differences in the Stress Response and in Cognitive Flexibility,” Biological Psychiatry 81, no. 8 (2017): 683–692. 27955897 10.1016/j.biopsych.2016.10.013 PMC5359079

[32] K. Sasaki , M. Suzuki , M. Mieda , N. Tsujino , B. Roth , and T. Sakurai , “Pharmacogenetic Modulation of Orexin Neurons Alters Sleep/Wakefulness States in Mice,” PLoS One 6, no. 5 (2011): e20360. 21647372 10.1371/journal.pone.0020360 PMC3103553

[33] D. Kunz , R. Mahlberg , C. Müller , A. Tilmann , and F. Bes , “Melatonin in Patients With Reduced REM Sleep Duration: Two Randomized Controlled Trials,” Journal of Clinical Endocrinology and Metabolism 89, no. 1 (2004): 128–134. 14715839 10.1210/jc.2002-021057

[34] L. Demisch , K. Demisch , and T. Nickelsen , “Influence of Dexamethasone on Nocturnal Melatonin Production in Healthy Adult Subjects,” Journal of Pineal Research 5, no. 3 (1988): 317–322. 3404401 10.1111/j.1600-079x.1988.tb00657.x

[35] W. S. Bahnasy , Y. A. E. El‐Heneedy , A. M. El‐Shamy , M. Y. Badr , R. A. Amer , and I. S. E. Ibrahim , “Sleep and Psychiatric Abnormalities in Gullian Barré Syndrome,” Egyptian Journal of Neurology, Psychiatry and Neurosurgery 54, no. 1 (2018): 5. 29780225 10.1186/s41983-018-0007-1 PMC5954782

[36] M. Ogasawara , M. Takeshima , S. Kosaka , et al., “Exploratory Validation of Sleep‐Tracking Devices in Patients With Psychiatric Disorders,” Nature and Science of Sleep 15 (2023): 301–312. 10.2147/NSS.S400944 PMC10143764 37123093

[37] K. Kanemura , H. Kadotani , M. Matsuo , et al., “Evaluation of a Portable Two‐Channel Electroencephalogram Monitoring System to Analyze Sleep Stages,” Journal of Oral & Sleep Medicine 2, no. 2 (2016): 101–108.

