# Supplementary material for: Sleep Architecture Alterations Following High‐Dose Steroid Pulse Therapy: A Pilot Study Using a Portable Electroencephalogram‐Based Device
Source: Neuropsychopharmacol Rep. 2025 Nov 9;45(4):e70071. doi: 10.1002/npr2.70071 (PMC12597610; doi:10.1002/npr2.70071)
Supplement: Supplementary file 1 — Figure S1: npr270071‐sup‐0001‐Figure S1.docx. [file NPR2-45-e70071-s001.docx]

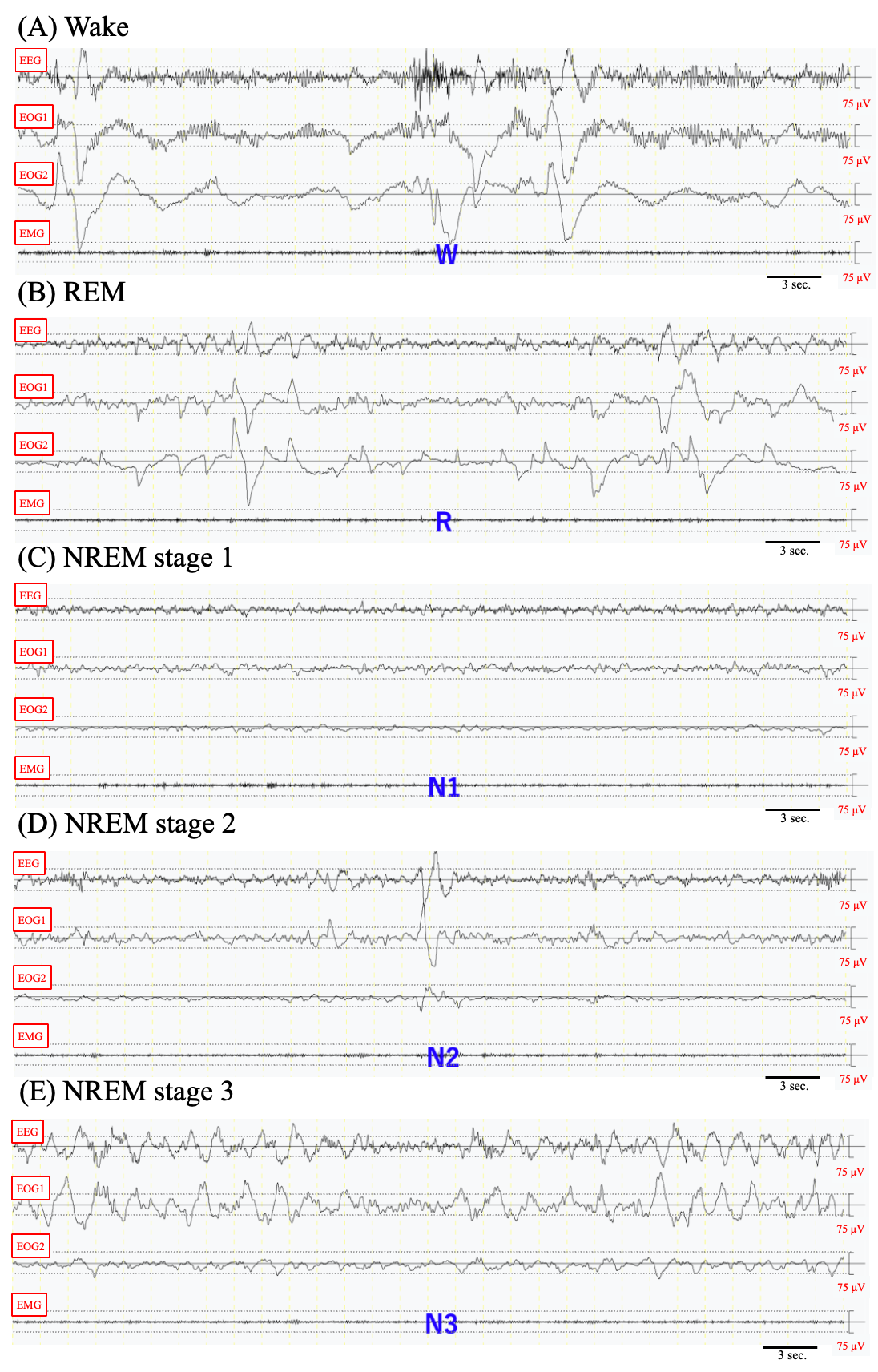


Supplementary Figure S1. Typical polysomnographic data

A representative 30-s epoch of four-channel recordings, consisting of electroencephalogram (EEG), two electro-oculogram (EOG1, EOG2), and chin surface electromyogram (EMG), is shown. The amplitude scale is ±75 µV. Panels A–E illustrate typical waveforms corresponding to Wake (A), REM sleep (B), NREM stage 1 (C), NREM stage 2 (D), and NREM stage 3 (E).
